# Hungarian general practitioners’ attitude and the role of education in dementia care

**DOI:** 10.1017/S1463423619000203

**Published:** 2019-07-01

**Authors:** Szilvia Heim, Csilla Busa, Éva Pozsgai, Ágnes Csikós, Edina Papp, Magdolna Pákáski, János Kálmán, Ferenc Hajnal, Kázmér Karádi

**Affiliations:** 1 Institute of Primary Health Care, Medical School, University of Pécs, Pécs, Hungary; 2 Department of Psychiatry, Faculty of Medicine, University of Szeged, Szeged, Hungary; 3 Department of Family Medicine, Faculty of Medicine, University of Szeged, Szeged, Hungary; 4 Institute of Behavioral Sciences, Medical School, University of Pécs, Pécs, Hungary

**Keywords:** attitude, dementia, education, general practitioners, Hungary

## Abstract

**Background:**

Dementia in the elderly constitutes a growing challenge in healthcare worldwide, including Hungary. There is no previous report on the role of general practitioners in the management of dementia.

**Aim:**

The purpose of the present study was to investigate the Hungarian general practitioners’ attitude toward their patients living with dementia as well as dementia care. Our goal was also to assess their willingness and habits in assessing dementia. Additionally we wanted to explore the role of education about dementia, and its impact on their attitude in dementia management.

**Methods:**

As part of a large survey, a self-administered questionnaire was filled out voluntarily by 402 of general practitioners. According to our preset criteria, 277 surveys were selected for evaluation. Descriptive statistical analysis and Likert-scale analysis were performed.

**Findings:**

Half of the doctors (49.8%) indicated that they conducted a test to assess cognitive functions in case of suspicion. Among the respondents who did not assess, 50.0% of physicians cited lack of time as the main reason for not doing so and 14.4% of them had not proper knowledge of testing methods. The respondents most often mentioned feelings toward their patients with dementia, were regret (Likert-scale mean: 3.33), helplessness (3.28) and sadness (3.07). The majority of physicians thought the treatment of dementia was difficult (4.46). Most of the respondents (81.2%) indicated that in the past 2 years they had not participated in any training about dementia. Those practitioners who had participated in some form of education were less likely to feel helpless facing a patient with dementia, and education also determined their approach to dementia care.

## Introduction

The population over 65 years of age is increasing in the world. Therefore, the prevalence and cost of dementia care is also going up. This puts even greater burden on less affluent countries, including Hungary (ADI). According to epidemiological studies in Hungary, the number of patients living with dementia is estimated between 100 000 and 500 000. Moreover, this number may double by 2050 (Érsek *et al*., 2010a). Dementia constitutes one of the main causes of disability in the elderly. According to the Global Burden of Disease Perspective, it takes 11.2% of all years lived with disability, which is higher than stroke (9.5%), heart disease (5%), and cancer (2.4%) (WHO). Due to lack of adequate epidemiological data, we have only estimates on disease burden and cost of dementia in Hungary (Érsek et al., 2010b).

Primary health care is in the first line in patient care. Nevertheless, compared to the management to other chronic diseases, dementia care constitutes only a small proportion of a general practitioner’s (GPs) workload, although the increasing aging population will change this situation in the future (Iliffe *et al*., 2009). The most common cause of dementia is Alzheimer’s disease which is 50–70 % of all cases (Ott *et al*., 1995). Exact data on the different types of dementia are not available for Hungary (Érsek *et al*., 2010a).

A number of studies have shown that dementia in elderly often (up to 80%) remains unrecognized in primary care (Boise *et al*., 2004; Connolly *et al*., 2011; Lang *et al*., 2017). Dementia, in general, is underdiagnosed and undertreated by GPs, in at least half of the patients over 65 (Boustani *et al*., 2005; Iliffe *et al*., 2009). They have especially difficulties in recognizing mild dementia (Mitchell *et al*., 2011). Various barriers have been identified as the cause of missing the early detection, which includes lack of time and financial constraints, doubts about the importance of assessment or diagnostic uncertainty. Hesitation concerning the efficacy of treatment or concerns about the emotional effect on the patient by disclosing the diagnosis may also be obstacles (Boise *et al*., 1999; Iliffe *et al*., 2005; Pimlott *et al*., 2009b; Koch and Iliffe 2010a; Lahjibi-Paulet *et al*., 2012; Caruana-Pulpan and Scerri, 2014; Gove *et al*., 2016). One of the most often mentioned obstacles in the early screening of dementia is the lack of knowledge regarding Alzheimer’s disease and other forms of dementia (Barrett *et al*., 1997; Koch *et al*., 2010b; Pathak and Montgomery, 2015).

In many countries (France, Netherlands, Norway, Cyprus, United Kingdom), a National Dementia Plan is available, where GPs have a pivotal role, especially in recognizing the early symptoms and participating in the coordination of care for people with dementia (Alzheimer-Europe). Despite this well-defined gate-keeper role, the activity of GPs varies and their perceptions and attitudes toward patients living with dementia greatly influence the management of these patients. Many studies shed light on the role of the attitude of GPs, their frustration, and lack of confidence in dementia care and management (Turner *et al*., 2004; Pathak and Montgomery, 2015; Subramaniam *et al*., 2018). An Irish study showed that GPs were retained from making the diagnosis of dementia due to lack of confidence and concerns about the impact of the diagnosis on the patient’s life (Cahill *et al*., 2006; 2008). According to another investigation, English GPs were worried about stereotypes linked to dementia, which cause difficulties in communication with people living with dementia (Gove *et al*., 2017). One French and an English analysis mention stigmatization of patients with dementia as the main barrier to setting up a diagnosis (Lahjibi-Paulet *et al*., 2012; Gove *et al*., 2016). According to a Canadian study, GPs have strong concerns about the role of the pharmaceutical industry on dementia guidelines (Pimlott *et al*., 2009a). This belief may affect GPs attitude in dementia care, as well.

Although Petrazzuoli *et al*. (2017) performed an informant survey of GPs’ attitudes regarding dementia management in 25 European countries including Hungary; however, the number of primary care physicians questioned per country was very small (Petrazzuoli *et al*., 2017). Until now there was no investigation in a larger population of GPs about their attitudes in the diagnosis and management of patients with dementia in Hungary.

As part of a larger project, the main aim of the present study was to investigate the Hungarian GPs’ attitude toward their patients living with dementia as well as dementia care. Our goal was also to examine their willingness and habits in assessing dementia. Additionally we wished to explore the role of education about dementia, as well as its modifying effect on GPs attitude in dementia management.

## Methods

### Setting and sampling

The present survey was carried out during a 6 months period of time in Hungary, with voluntarily participating GPs who were attending obligatory, postgraduate training courses in family medicine or national conferences for GPs. During the time of our survey and in earlier period, none of the obligatory, postgraduate training programs for GPs contained material on dementia. The only opportunities for GPs to acquire skills and knowledge about dementia were by individual learning or at symposia organized by pharmaceutical companies. The inclusion criteria specified that respondents have to provide direct patient care. Participation was anonymous and there was no financial compensation for taking part in the survey. GPs from all regions of Hungary’s participated.

### Questionnaire

As part of a more extensive research project, the research team developed a self-evaluation questionnaire to investigate the major aspects of dementia care in the Hungarian primary care system. The aim of the *‘General practitioners’ attitudes regarding the management of dementia in primary care’* project was to obtain information about GPs’ attitude, habits, knowledge, and personal experiences in recognizing and caring for dementia patients in Hungary.

Our study aimed to focus on the following: (1) Investigate GPs’ attitude toward their patients living with dementia and dementia care; (2) examine GPs’ testing habits in the case of suspected cognitive impairment; (3) acquire information about GPs’ participation in any kind of educational programs on dementia; and (4) analyze the impact of participation in dementia education on modifying GPs’ attitudes toward dementia care. For this purpose, in this study 10 questions were selected for further analysis. Other parts of the research project will be published elsewhere. Four hundred and two questionnaires were collected from participant GPs. In line with our objectives, only those questionnaires were included for statistical analysis where the feelings and education related questions were fully answered. Finally, 277 questionnaires were used for this analysis.

Four questions pertained to the general characteristics of the GPs (age, gender) and their practices (location, number of patient visits). Six questions were intended to investigate the feelings, testing habits related to the recognition and management of patients living with dementia as well as participation in any kind of education about dementia. These include two single dichotomous (yes/no) items, two multiple-response items, and two 5-point Likert-scale questions.

### Statistics

Statistical analysis was undertaken using IBM SPSS Statistics for Windows, version 24.0 statistical program. We performed descriptive analysis, including frequency distribution tables and crosstabs, and analysis of variance. Statistical significance was assessed by Fisher’s Exact test and *t*-test. *P* < 0.05 was considered statistically significant.

## Findings

The demographic profile of the respondents is shown in Table 1. Among them, we observed an equal gender split (137 females and 130 males, with 10 missing) and a 2/3 urban, 1/3 rural split. Almost half (48.7%) of the participating GPs were above 55 years of age. Only a small percentage (18.4%) of participants had less than 40 patient-visits per day and 27.4% of the GPs had more than 60 patient visits on average per day.

**Table 1.** Selected demographic characteristics of respondents

Sampled respondent characteristics	Respondents **n** (%)
**Practice location**	
Capital city	**33** (11.9)
Urban	**143** (51.7)
Rural	**86** (31.0)
Missing answer	**15** (5.5)
**GPs’ age (years)**	
25–35	**24** (8.7)
36–45	**41** (14.8)
46–55	**74** (26.7)
56–65	**100** (36.1)
66 <	**35** (12.6)
Missing answer	**3** (1.1)
**Gender**	
Male	**130** (46.9)
Female	**137** (49.5)
Missing answer	**10** (3.6)
**Number of patient-visits per day**	
< 30	**4** (1.4)
30–40	**47** (17.0)
40–50	**72** (26.0)
50–60	**73** (26.4)
60 <	**76** (27.4)
Missing answer	**5** (1.8)

Approximately half of the GPs (49.8%) indicated that they performed the assessment of cognitive functions in patients where they suspected cognitive function impairment. Hundred and thirty-two respondents (47.7%) reported that they did not use any tests even if the suspicion of cognitive impairment arose. Of the 138 participants who did perform tests, 81.2% used the Clock Drawing Test, 58.7% used the MMSE Test and 56.5% the orientation-questions. Examination of writing was used by 26.1% of these respondents, while the other specific tests were used only by 2.9% of them. When respondents were questioned about the reasons underlying their disinclination to assess, 50.0% of GPs cited lack of time as the main reason and 14.4% of GPs said that they had not knowledge about any kind of testing method. Other reasons for not testing included: testing should be done by a specialist (8.3%) and testing for cognitive functions is not important (4.5%). A total of 17.4% of the GPs did not provide an answer (Table 2).

**Table 2.** GPs propensity to conduct cognitive testing in case of suspicion for dementia and reasons behind not testing

Do you use a special test for testing cognitive functions if you suspect cognitive impairment in your patient?	Responses n (%)
Yes	**138** (49.8)
No	**132** (47.7)
Missing answer	**7** (2.5)
**Which instrument do you use in your everyday work to assess the severity of dementia?** (You may sign more answers.) **N = 138**	
Mini-Mental State Examination	**81** (58.7)
Clock Drawing Test	**112** (81.2)
Writing examination	**36** (26.1)
Questions about time/place orientation	**78** (56.5)
Another special screening tool	**4** (2.9)
Missing answer	**2** (1.4)
**If you do not use a special test to assess cognitive impairment, what is your reason for not doing so?** **N = 132**	
I am not aware that such a test is available	**19** (14.4)
I do not have enough time	**66** (50.0)
I do not find it important to test	**6** (4.5)
Loss of memory is a natural age-related process	**2** (1.5)
I do not want to frighten the patient and his/her relatives	**2** (1.5)
I will check it six months later	**3** (2.3)
Testing should be done by specialists	**11** (8.3)
Missing answer	**23** (17.4)

Two survey items were designed to analyze the prevailing attitudes of Hungarian GPs regarding the perceptions about their patients living with dementia and their treatment. The feelings that may arise in physicians when taking care of patients with dementia were analyzed by the Likert scale. The most often reported feelings while managing these patients were regret (3.33), helplessness (3.28), and sadness (3.07) (Table 3).

**Table 3.** Respondents’ quality of feelings when caring for patients living with dementia (Respondent needed to indicate whether he/she agreed with the feeling on a scale of 1 to 5, where 1 is ‘Not at all’ and 5 is ‘I agree completely’.)

	Shock	Feel bad	Regret	Helplessness	Sadness	Doubtfulness
**N** – valid Missing	277	277	277	277	277	277
	0	0	0	0	0	0
**1 – not at all**	18.8%	33.2%	11.2%	12.6%	14.4%	24.5%
**2**	17.7%	21.7%	12.3%	14.4%	15.2%	22.4%
**3**	28.2%	27.1%	27.4%	25.3%	31.4%	33.2%
**4**	22.4%	14.1%	30.3%	27.4%	26.7%	15.2%
**5 – I agree completely**	13.0%	4.0%	18.8%	20.2%	12.3%	4.7%
**Mean**	**2.93**	**2.34**	**3.33**	**3.28**	**3.07**	**2.53**
**Std. deviation**	1.293	1.189	1.233	1.288	1.220	1.153

Respondents were asked to rate their perception about the difficulty to treat dementia. The Likert-scale analysis (where 1 is Easy and 5 is Very difficult) demonstrated that the majority of GPs (89.6%) thought that the treatment of dementia was difficult or very difficult for them (Mean: 4.464, Std. deviation 0.835).

Of the 277 respondents, the majority (81.2%) of participants answered that they had not taken part in any type of education about dementia in the past 2 years. Only 18.8% of the GPs had participated in some form of dementia-related training.

Our data revealed that the group of GPs who participated in any form of education about dementia was inclined to assess significantly more patients (68.8%) for cognitive impairment in case of suspicion. Those who had not received any education in the past 2 years assessed fewer patients (47.3%) (Table 4).

**Table 4.** Association between education and propensity to test for cognitive impairment

		Education		Total
		YES	NO	
**Propensity to test**	YES	68.8%	47.3%	51.1%
	NO	31.3%	52.7%	48.9%
	Total	100.0%	100.0%	100.0%

(Fisher’s Exact test, *P*-value = 0.005)

Participant GPs indicated regret (3.33) and helplessness (3.28) most often toward their patients living with dementia. The most often reported feeling was regret, proved to be dominant regardless of having participated or not in education about dementia. This appears to be generally experienced by GPs. However, helplessness, the second most often mentioned feeling was stronger among participants who had not taken part in any form of education about dementia (3.38) than in those who had some training (2.87) (Table 5).

**Table 5.** Association between participation in dementia-related education and feeling of helplessness

Helplessness	Mean	N	Std. deviation
Had training	2,87	52	1.299
No training	3.38	225	1.269
Total	3.28	277	1,288

(one-way ANOVA test, *P* = 0.009)

## Discussion

The present report is the first part of a large-scale, dementia-related survey where Hungarian GPs’ role, habits, knowledge, and personal experiences related to dementia were examined. According to our results, only approximately half of the Hungarian GPs conduct a cognitive assessment of patients suspected of cognitive impairment. Lack of time and of knowledge were specified as the main obstacles. Over 80% of respondents had not received any form of education about dementia in the previous 2 years. This may be the underlying cause that the GPs find the management of dementia very difficult. Furthermore, the sense of helplessness beside regret was a crucial attitude in their disposition toward their patients living with dementia.

Hungarian experiences about GPs’ activity in cognitive assessment is in contrast to a North-American report on the practice patterns and screening habits of GPs, where 93% of physicians scan or conduct diagnostic evaluations for dementia in older patients (Stewart *et al*., 2014). An Australian study has similar data, as it states that the vast majority of GPs conduct the formal cognitive assessment using a validated test (e.g., MMSE) in individuals with suspected cognitive impairment (Murphy *et al*., 2014). On examining the cause of the omission of cognitive testing, the majority of Hungarian GPs cited the lack of time as the main reason. In Hungary, according to our data doctor–patient encounters with GPs often reach 40–60 or more patients/day. Time constraint felt by GPs is obvious; consequently, they allocate much shorter time to each patient. This is in agreement with previous studies, which demonstrated that GPs perceived time constraints was the major obstacle to good quality dementia care (Turner *et al*., 2004; Pimlott *et al*., 2009b; Koch *et al*., 2010b). On the other hand, more than a tenth of the GPs in our study answered that their reason for not testing was due to their lack of knowledge of cognitive assessment tools. This finding is in accordance with data from other countries (Barrett *et al*., 1997; Pucci *et al*., 2004; Cahill *et al*., 2006; Pathak and Montgomery, 2015; Veneziani *et al*., 2016). In two Italian studies, problems in recognizing early symptoms of Alzheimer’s disease and screening patients have been linked to the lack of specific training (Pucci *et al*., 2004; Veneziani *et al*., 2016). According to an investigation conducted in Ireland, 90% of GPs had never undergone any type of dementia-specific training (Cahill *et al*., 2006).

In our analysis, we found that the group of GPs who participated in any type of education about dementia was significantly more likely to perform the cognitive assessment (68.8%) than those who did not (47.3%). This is also important because the active participation of GPs in the early detection of dementia ensures better treatment, access to psychosocial and pharmacological interventions and leads to improved cost-effectiveness (Barnett *et al*., 2014).

Helplessness is a frequent feeling felt by GPs and it was more prevalent among participants who had not undergone in any form of education about dementia. This view may inhibit them in taking on an active, supportive role. This finding is important because the feeling of helplessness may reduce their confidence and hamper their activity in helping their patients. In contrast, GPs who had participated in education about dementia in the past 2 years were less likely to feel a sense of helplessness, so they can play a more active role in the management of their patients with dementia. These findings draw attention to the importance to further education about dementia of Hungarian GPs.

Many studies throughout the world have reported the need for special, broad-scale, and regular training programs for GPs (Koch *et al*., 2010b; Veneziani *et al*., 2016; Dreier-Wolfgramm *et al*., 2017). The importance of training cannot be emphasized enough to achieve higher quality dementia care. Key factors enabling GPs to conduct cognitive assessment must have an awareness of the need to carry out a cognitive examination; possessing the necessary skills and confidence; and having adequate time and resources (Murphy *et al*., 2014). The specific training in the field of dementia is emphasized in the literature. Dementia-trained physicians had significantly higher confidence than the non-trained (Liu *et al*., 2013). According to the Recaredem study, GPs with higher confidence are more prepared to carry out specific actions to manage dementia (Harmand *et al*., 2018). Many studies confirm the importance of training programs for GPs as it improves the rates of reported dementia cases and achieves better participation in the management of dementia (Downs *et al*., 2006; Caruana-Pulpan and Scerri, 2014). Training and support for GPs may change their attitude that they have little to offer to patients living with dementia and to their relatives (Downs, 1996). Several studies confirm that GPs’ training should be practical, evidence-based and relevant, as well as tailored to their special needs: take into account their maturity, using individualized methods and include interprofessional education (Iliffe *et al*., 2002; Dreier-Wolfgramm *et al*., 2017).

These findings imply that the training of Hungarian GPs may result in improved cognitive assessment of their patients and may change the perception about their role in dementia care. In Hungary, special training programs for GPs should be urgently developed and tailored to their needs.

Our study has some limitations. First, our sampling process was not selected from an official list of the Hungarian GPs and completing the survey was voluntary, which may have altered the results. Second, according to our selection criteria, we excluded 125 incomplete questionnaires from the 402 we collected. Third, a possible reason for not completing the questionnaire may have been a lack of interest in the subject of dementia. Therefore, the results may represent a more favorable approach to assessing dementia than what may actually exist. Fourth, the survey was a self-report on patterns of practice and attitude toward patients with dementia. We cannot know whether the results truly reflect the actual practice patterns of Hungarian GPs regarding dementia.

## Conclusion

Our study demonstrated that Hungarian GPs’ present attitude was an important obstacle in the effective assessment of patients living with dementia. We recognized the deficiency of and the need for dementia education for Hungarian GPs. Postgraduate training of GPs in the field of dementia may open the opportunity for them to play a more active role in the management of dementia. This requires the development and the implementation of educational programs in Hungary as well as further studies on its effect on GPs’ attitude and activity in dementia care.

Our results signify the need for a unified dementia plan for Hungary, where the role of GPs is defined in the management of dementia. Time constraints are alleviated by multidisciplinary teams and GPs are offered training and provided with straightforward guidelines. With implementing such measures in primary practice, an improvement in the quality of dementia care can be expected.
